# Stable Cu Catalysts Supported by Two‐dimensional SiO_2_ with Strong Metal–Support Interaction

**DOI:** 10.1002/advs.202104972

**Published:** 2022-01-25

**Authors:** Shenghua Wang, Kai Feng, Dake Zhang, Deren Yang, Mengqi Xiao, Chengcheng Zhang, Le He, Binhang Yan, Geoffrey A. Ozin, Wei Sun

**Affiliations:** ^1^ State Key Laboratory of Silicon Materials School of Materials Science and Engineering Zhejiang University Hangzhou Zhejiang 310027 P. R. China; ^2^ Department of Chemical Engineering Tsinghua University Beijing 100084 China; ^3^ Institute of Functional Nano and Soft Materials (FUNSOM) Jiangsu Key Laboratory for Carbon‐Based Functional Materials and Devices Soochow University Suzhou Jiangsu 215123 China; ^4^ Materials Chemistry and Nanochemistry Research Group Solar Fuels Cluster Departments of Chemistry University of Toronto Toronto Ontario M5S 3H6 Canada

**Keywords:** Cu/SiO_2_, high‐temperature stability, strong metal–support interaction (SMSI)FF

## Abstract

Cu‐based catalysts exhibit excellent performance in hydrogenation reactions. However, the poor stability of Cu catalysts under high temperatures has restricted their practical applications. The preparation of stable Cu catalysts supported by SiO_2_ with strong metal–support interaction (SMSI) has thus aroused great interest due to the high abundance, low toxicity, feasible processability, and low cost of SiO_2_. The challenge in the construction of such SMSI remains to be the inertness of SiO_2_. Herein, a simple and scalable method is developed to prepare 2D silica (2DSiO_2_) supported Cu catalysts with SMSI by carefully manipulating the topological exfoliation of CaSi_2_ with CuCl_2_ and thereafter calcination. The prepared Cu‐2DSiO_2_ catalysts with the unique encapsulated Cu nanoparticles exhibit excellent activity and long‐term stability in high‐temperature CO_2_ hydrogenation reactions. This feasible and low‐cost solution for stabilizing Cu catalysts might shed light on their realistic applications.

## Graphical abstract

Achieving long‐term stability of Cu/SiO_2_ catalysts at high temperatures has been a long‐sought‐after goal for many catalytic reactions that demand low cost and high throughput. Herein, a cost‐effective and feasible route is developed to prepare a new Cu/2DSiO_2_ catalyst with strong metal–support interaction that exhibits ultrahigh stability at high temperatures, which is not enjoyed by the traditional impregnation method.

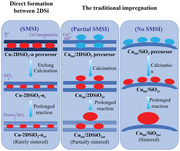

## 1 Introduction

Cu‐based catalysts have been tremendously studied in hydrogenation reactions as Cu exhibits prominent activity in the dissociation of H_2_ to generate highly active H that could easily reduce the reactants.^[^ ^1^, ^2^, ^3^, ^4^, ^5^, ^6^, ^7^, ^8^, ^9^, ^10^, ^11^, ^12^ ^]^ When it comes to the hydrogenation of carbon–oxygen species, Cu^+^ species could also act as effective promoters by polarizing the carbon–oxygen bonds to activate the reactants.^[^ ^13^, ^14^, ^15^, ^16^ ^]^ However, deactivation commonly occurs for conventional Cu‐based catalysts via sintering of metal particles under high temperature and redox reactions of copper species of mutable valences (Cu^0^ and Cu^+^) in the strongly reducing or oxidizing atmospheres.^[^ ^17^, ^18^, ^19^ ^]^ This has restricted the industrial implementations of these Cu‐based catalysts.

The exploration of stable Cu catalysts supported by SiO_2_ has aroused tremendous interest due to the high abundance, low toxicity, feasible processability, and low cost of SiO_2_.^[^ ^20^, ^21^, ^22^, ^23^, ^24^, ^25^, ^26^, ^27^, ^28^, ^29^, ^30^, ^31^, ^32^, ^33^, ^34^, ^35^ ^]^ However, reports regarding the construction of SMSI, a widely practiced and effective means to stabilize metals, are rare for Cu/SiO_2_ catalysts due to the relative inertness of SiO_2_ compared with reducible oxides like CeO*_x_*, TiO*_x_*, etc.^[^ ^36^ ^]^ Classical SMSI refers to the physical coverage/encapsulation of metal nanoparticles with oxide overlayers to prevent the migration.^[^ ^37^, ^38^, ^39^, ^40^, ^41^ ^]^ To aid the encapsulation by SiO_2_, previous arts regarding the synthesis of stable Cu/SiO_2_ catalysts with SMSI required evaporation of the corrosive ammonia, careful regulation of pH, and surfactants.^[^ ^36^, ^42^, ^43^ ^]^ Moreover, most of these Cu catalysts were tested at mild temperatures below 350 ℃, so their stability for those reactions performed at high temperature and high conversion is still a mystery. Therefore, it still remains a challenge to design feasible and low‐cost processes to prepare Cu/SiO_2_ catalysts with SMSI and thereby stable catalytic activity, especially under high temperature.

Herein, we proposed a new strategy to prepare 2DSiO_2_ supported Cu catalysts (Cu‐2DSiO_2_) with high stability. This Cu‐2DSiO_2_ catalyst was obtained by tuning the topological exfoliation chemistry of CaSi_2_ with CuCl_2_ which directly dispersed Cu nanoparticles between 2D silicon nanosheets (2DSi), followed by calcination to transform the composites into active Cu‐2DSiO_2_ catalysts. The endothermic CO_2_ hydrogenation to CO was utilized as a model reaction to determine the catalytic performance of the prepared Cu catalyst at high working temperatures. The Cu‐2DSiO_2_ catalysts exhibited superior activity and stability during a prolonged test in comparison to control catalysts prepared through the traditional impregnation method. We demonstrate that the unique Cu–O–Si encapsulation generated from SMSI are responsible for the superiority of these Cu‐2DSiO_2_ based samples. This new Cu on SiO_2_ catalyst that can be prepared in large quantity and with low cost sheds light on its practical applications.

## 2 Results

### 2.1 Characterization of the Catalysts

**Figure 1:**
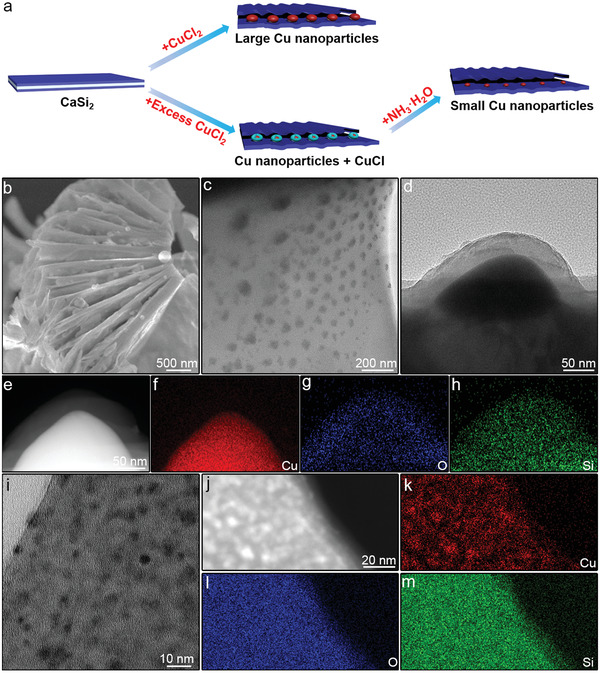
a) Schematic of the synthesis process of Cu‐2DSiO_2_ based catalysts. The blue sheet, the white sheet, the red nanoparticles, and the cyan shell around red particles are composed of Si, Ca, Cu, and Cu^+^, respectively. b) SEM image, and c) TEM image of 2DSi supported large Cu nanoparticles. d) TEM image, and e–h) EDS mappings of 2DSiO_2_ supported large Cu nanoparticles after calcination. i) TEM image, and j–m) EDS mappings of Cu‐2DSiO_2_‐400_r_.

Recently CuCl_2_ has been reported to exfoliate CaSi_2_ into Si nanosheets, which demonstrated decent performances as anodes for Li‐ion batteries.^[^ ^44^ ^]^ The excess amount of CuCl_2_ was used as the sacrificial reagent, and discarded after isolation of 2DSi. In contrast, as demonstrated in **Figure** **1**a, we prepared samples that preserved Cu between the 2D Si nanosheets via the exfoliation of CaSi_2_ with the designed amount of CuCl_2_. We discovered that the category of the Cu species obtained (Cu, Cu^+^ or the mixture of both) depended on the concentration of the CuCl_2_ precursor (*C*_0_) when the fixed amounts of CaSi_2_ and solvent were used. First, when *C*_0_ equals 0.2 m, Cu nanoparticles with relatively large size (60 nm) supported by 2DSi could be obtained through the reaction of CaSi_2_ + CuCl_2_ → Cu + 2Si + CaCl_2_ (Figure 1b,c and Figure S1, Supporting Information). To investigate whether SMSI could be formed between Cu and 2DSi, the solid products were collected and then calcined under air to remove any adsorbed solvent and enhance the interaction between the Cu species and the support, followed by reduction in H_2_ to recover the active Cu^0^ species. It is surprising to observe that a layer of silica could grow on the surface of the Cu nanoparticles after this calcination process (Figure 1d–h). This encapsulation structure is a distinctive feature of the successful construction of SMSI, which can improve the high‐temperature stability of these samples of Cu encapsulated by 2DSiO_2_. However, such large nanoparticles usually do not qualify for high catalytic activities and stabilities.^[^ ^45^, ^46^, ^47^ ^]^ Notably, when excess amounts of CuCl_2_ (*C*_0_ > 0.2 m) are added to the reactants, the as‐formed copper nanoparticles are etched through a comproportionation reaction (Cu + CuCl_2_ → 2CuCl). This etching strategy was used as an effective method to reduce the size of Cu by carefully controlling the amount of CuCl_2_ precursor. Partially etched Cu nanoparticles supported by 2DSi could be obtained when appropriate amounts of CuCl_2_ were used as the precursor, shown in Figure 1a. This could be corroborated by the X‐ray diffraction (XRD) results showing the decreased and increased signals for metallic copper and CuCl, respectively, as *C*_0_ grows, suggesting the reduced proportion of Cu (0) species (Figure S2, Supporting Information). 0.2085 m was found to be a critical value for *C*_0_ to keep the coexistence of Cu and CuCl, above which all Cu (0) transformed into CuCl. Therefore, 0.2085 m was selected as the appropriate *C*_0_ for the preparation of small‐sized copper nanoparticles encapsulated by 2DSi. The remnant by‐product CuCl was removed by NH_3_·H_2_O. The solid products (Cu‐2DSi) were collected and then calcined at 400 ℃ under air (denoted as Cu‐2DSiO_2_‐400). The Cu‐2DSiO_2_‐400 precursor exhibited a layered structure (Figure S3a, Supporting Information). Notably, the size of CuO nanoparticles in Cu‐2DSiO_2_‐400 (4 nm) is much smaller than that of the unetched Cu nanoparticles obtained using 0.2 m CuCl_2_ (60 nm), shown by Figure S3b, Supporting Information, Figure 1c and Figure S1 (Supporting Information). This is beneficial for the later transformation of CuO into the active catalyst with highly dispersed copper. Energy dispersive spectrometer (EDS) mappings demonstrated that Cu is distributed throughout the stack formed by Si nanosheets (Figure S3c–f, Supporting Information). The Cu‐2DSiO_2_‐400 precursor was further reduced at 500 ℃ under H_2_ to be transformed into an active Cu catalyst (the corresponding sample is denoted as Cu‐2DSiO_2_‐400_r_). XRD results suggest that the CuO species has transformed into metallic copper (Figure S4, Supporting Information). The Cu nanoparticles maintained high dispersion with an average diameter of 3 nm after reduction (Figure 1i and Figure S5a, Supporting Information). EDS mappings showed that Cu, Si, and O were distributed throughout the 2D structure (Figure 1j–m). These results suggest that the etching and calcination procedures are indispensable for the construction of Cu‐2DSiO_2_ based catalysts with small Cu nanoparticles.

For comparison, two control samples were prepared by impregnation of Cu(NO_3_)_2_·3H_2_O on 2DSi and a commercial SiO_2_ support, respectively. These two samples were annealed at 850 ℃ in air, followed by reduction at 500 ℃ in H_2_. The corresponding samples were denoted as Cu_im_/2DSiO_2r_ and Cu_im_/SiO_2r_, respectively. The copper nanoparticles were in the metallic states in both samples, confirmed by the XRD results (Figure S6, Supporting Information). In contrast to the relatively homogeneous and small size distribution of Cu‐2DSiO_2_‐400_r_, large aggregates were frequently found for these two samples (Figures S7a and S8a, Supporting Information), suggesting the advantages of the strategy illustrated in Figure 1a in the preparation of Cu catalysts with highly dispersed copper.

### 2.2 Thermal Catalytic Performance

**Figure 2:**
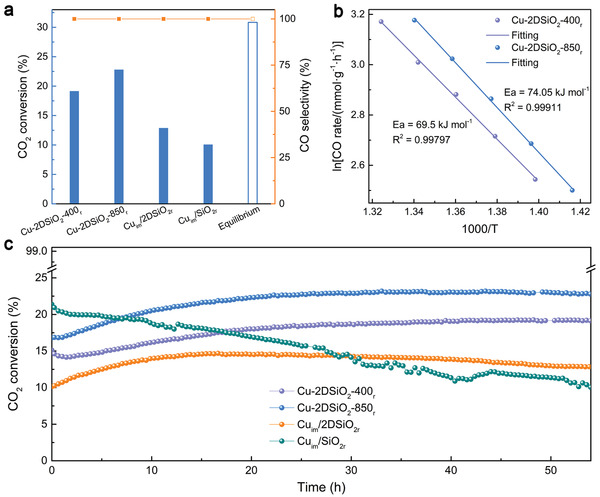
Catalytic performance of CO_2_ hydrogenation (mass of the catalyst: 50 mg; reaction temperature: 500 ℃; flow rates of the reactants: CO_2_/H_2_/N_2_ = 5/5/5 mL min^‐1^). a) CO_2_ conversion after 54 h testing, and CO selectivity. The hollow column in (a) represents the thermodynamical equilibrium CO_2_ conversion for reverse water‐gas shift reaction at 500 ℃ when the flow rate of CO_2_ equals that of H_2_. b) Arrhenius plot fitting from the CO rates (Table S2, Supporting Information). c) CO_2_ conversion under 500 ℃ during a 54 h test for the different Cu catalysts.

As shown above, the Cu‐2DSiO_2_ based catalyst possesses highly dispersed Cu nanoparticles supported by layered 2DSiO_2_, which should render it an excellent candidate for many catalytic reactions that can be driven by Cu‐based catalysts. Herein, we selected CO_2_ hydrogenation as a model reaction to evaluate the catalytic performance of the prepared Cu catalysts, since Cu is known to be highly active for the endothermic reversed water gas shift (RWGS) reaction which favors high temperature and thereby requires high thermal stability of the catalyst. As shown in **Figure** **2**a, all the Cu catalysts exhibited 100% selectivity towards CO, but Cu‐2DSiO_2_‐400_r_ exhibits a much higher CO_2_ conversion (19.1%) than Cu_im_/2DSiO_2r_ (12.9%) and Cu_im_/SiO_2r_ (10.1%) with the same loading amount of Cu. This should be attributed to the much smaller size of the Cu nanoparticles in Cu‐2DSiO_2_‐400_r_ in comparison to Cu_im_/2DSiO_2r_ and Cu_im_/SiO_2r_. Furthermore, the superiority of Cu‐2DSiO_2_ based catalysts could be further improved to as high as 22.7% (35.9 mmol g^–1^ h^–1^), by enhancing the calcination temperature under air from 400 ℃ to 850 ℃ (the corresponding sample was denoted as Cu‐2DSiO_2_‐850). After the same reduction treatment at 500 ℃ under H_2_ (denoted as Cu‐2DSiO_2_‐850_r_), the size of the Cu nanoparticles (6.48 nm, Figure S9b, Supporting Information) was still much smaller than that of the unetched Cu nanoparticles (60 nm), but grew slightly larger compared with Cu‐2DSiO_2_‐400_r_ (3 nm). Therefore, the higher CO_2_ conversion of Cu‐2DSiO_2_‐850_r_ (22.8%) than Cu‐2DSiO_2_‐400_r_ (19.1%) cannot be attributed to the size difference. According to the Arrhenius plots, the Cu‐2DSiO_2_‐400_r_ and Cu‐2DSiO_2_‐850_r_ catalysts showed similar apparent activation energy for CO production, suggesting the same reaction mechanism occurred on these two samples (Figure 2b). Therefore, the difference in the activity should origin from the different number of active sites, which will be further discussed in the next section. Notably, the high catalytic performance of Cu‐2DSi‐850_r_ was further manifested by tuning the space velocity, gas ratio, and reaction temperature. As shown in Table S1 (Supporting Information), A high CO_2_ conversion of 38% (space velocity: 18 840 mL g_cat_^–1^ h^–1^, CO_2_:H_2_:N_2_ = 1:4:1.28, 500 ℃) or CO rate of 4442 mmol g_Cu_^–1^ h^–1^ (space velocity: 3 000 000 mL g_cat_^–1^ h^–1^, CO_2_:H_2_:N_2_ = 1:2:2, 550 ℃) was obtained, which is comparable to the recently reported high‐performance Cu catalysts for RWGS reaction.^[^ ^48^, ^49^ ^]^

The long‐term stability of the Cu catalysts was further evaluated in the flow reactor (Figure 2c). The values of CO_2_ conversion of all the Cu catalysts were below the equilibrium value under this testing condition, which guaranteed the stability property of these samples was valid (Figure 2a). Notably, a rising stage was only observed for the 2DSiO_2_ supported Cu catalysts (either the Cu‐2DSiO_2_‐400_r_ and Cu‐2DSiO_2_‐850_r_ samples or Cu_im_/2DSiO_2r_) which might be ascribed to the special metal–support interaction (MSI) between Cu and 2DSiO_2_. Particularly, Cu‐2DSiO_2_‐400_r_ exhibited a small drop in the conversion during the first 2 h, and then an everlasting rising stage, whereas Cu‐2DSiO_2_‐850_r_ exhibited almost no drop in conversion at any stage: a rising stage from the beginning, and then stabilized CO_2_ conversion after 33 h. TEM results reveal that the Cu nanoparticles of these two samples kept similar size distribution after the 54 h test (Figures S5 and S9, Supporting Information). In comparison, Cu_im_/2DSiO_2r_ exhibited poorer stability than Cu‐2DSiO_2_‐400_r_ and Cu‐2DSiO_2_‐850_r_. The degraded catalytic performance of Cu_im_/2DSiO_2r_ clearly resulted from the sintering of Cu after reaction (Figure S7, Supporting Information). The worst stability was observed for Cu_im_/SiO_2r_, which is also associated with the sintering of Cu (Figure S8, Supporting Information), typical for metal nanoparticles without structural engineering. Besides, our Cu‐2DSiO_2_ based catalysts exhibit obviously high stability in comparison to many reported Cu catalysts for RWGS (Table S3, Supporting Information). Instead of activity loss seen in other references, our catalysts exhibited activity gain. These results demonstrate that the 2DSiO_2_ structure can effectively stabilize Cu under the high temperature catalytic environment, and that our method of growing Cu nanoparticles directly between the silica nanosheets can further prevent sintering compared with the traditional impregnation method.

### 2.3 The Origin of the Enhanced Activity and Excellent Stability

**Figure 3:**
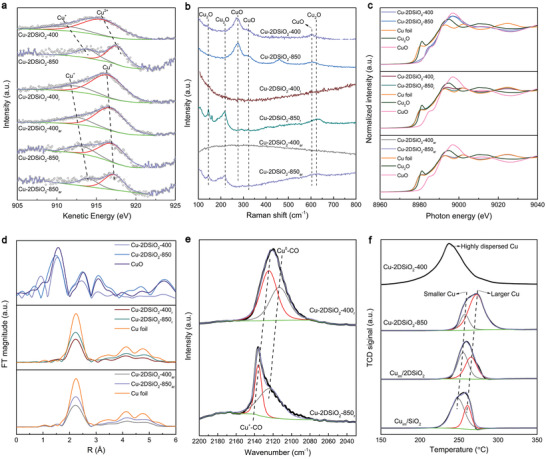
a) Cu LMM XAES spectra, b) Raman spectra, c) Cu K‐edge XANES spectra, d) the Fourier transform curves of the EXAFS spectra at the Cu K‐edge, e) diffuse reflectance Fourier transform infrared spectroscopy (DRIFTS) spectra of CO adsorbed at room temperature and f) temperature‐programmed reduction (TPR) profiles of different Cu catalysts.

To study the origin of the enhanced activity and excellent stability of the Cu‐2DSiO_2_ based catalysts in contrast to Cu_im_/2DSiO_2r_ and Cu_im_/SiO_2r_, a series of characterizations were performed to reveal the chemical structures of these Cu catalysts. First, X‐ray photoelectron spectroscopy (XPS) experiments were performed to study the surface chemical states. Cu 2p X‐ray photoelectron spectra (XPS) spectra show that two peaks of Cu 2p_1/2_ (953.6 eV) and Cu 2p_3/2_ (933.6 eV) with a satellite peak centered at around 943.2 eV exist for Cu‐2DSiO_2_‐850, indicative of the surface Cu^2+^ species of Cu‐2DSiO_2_‐850 (Figure S10, Supporting Information).^[^ ^19^ ^]^ Compared with Cu‐2DSiO_2_‐400, the peaks of CuO slightly shift to lower binding energy, suggesting the stronger metal‐support interaction of Cu‐2DSiO_2_‐850. The used catalysts after the 54 h reaction were denoted as Cu‐2DSiO_2_‐400_ar_, Cu‐2DSiO_2_‐850_ar_, Cu_im_/2DSiO_2ar_ and Cu_im_/SiO_2ar_, corresponding to Cu‐2DSiO_2_‐400, Cu‐2DSiO_2_‐850, Cu_im_/2DSiO_2_ and Cu_im_/SiO_2_, respectively. The reduced and used samples of Cu‐2DSiO_2_‐400 and Cu‐2DSiO_2_‐850 exhibit two main peaks of Cu 2p_1/2_ (952.2 eV) and Cu 2p_3/2_ (932.4 eV) with the disappearance of the satellite peak. These results indicate the Cu species in these two samples, which correspond to the actual active sites during the reaction, are Cu^+^ or Cu^0^.^[^ ^17^ ^]^ This was further verified by the X‐ray induced Auger electron spectra (XAES) of the Cu LMM region (**Figure** **3**a). The fitting results show two peaks corresponding to Cu^+^ and Cu^2+^ for Cu‐2DSiO_2_‐400 and Cu‐2DSiO_2_‐850. For the reduced and used samples, the Cu LMM XAES curves could be also deconvoluted into two peaks, but corresponding to Cu^+^ and Cu^0^, respectively. Therefore, the Cu^+^ always existed for the Cu‐2DSiO_2_ based catalysts under all three conditions: after oxidation in air, after reduction in H_2_, and after reaction in the H_2_:CO_2_ mixture. The fact that Cu^+^ did not fully transform into Cu^2+^ or Cu^0^, suggests strong metal‐support interaction of these samples. Moreover, both for the Cu‐2DSiO_2_‐400_r_ and the Cu‐2DSiO_2_‐850_r_ catalysts, the kinetic energy of either Cu^+^ or Cu^0^ increased from the reduced state to the used sample after the RWGS reaction, which should be ascribed to the reconstruction of the catalyst to generate more stable Cu–O–Si species during the reaction processes. This coincides with the increased catalytic activity in the initial stage and thereafter stable yield of CO product during the long‐time testing experiment. Notably, the kinetic energy of each Cu species (Cu^0^, Cu^+^ or Cu^2+^) for the Cu‐2DSiO_2_‐850 series is higher than that for the Cu‐2DSiO_2_‐400 series, suggesting the higher electronegativity of Cu species for the Cu‐2DSiO_2_‐850 sample. This should be ascribed to the stronger metal–support interaction for Cu‐2DSiO_2_‐850 through the formation of Cu–O–Si species. The unique Cu–O–Si species with SMSI were also verified by the Si 2p and O 1s XPS spectra (Figure S11, Supporting Information). It was found that the binding energies of Si and O for the Cu‐2DSiO_2_‐850 based samples were higher than those for the Cu‐2DSiO_2_‐400 based ones. To elucidate the main reason for this, two control samples were prepared by calcining bare 2DSi (obtained by removing the copper species in Cu‐2DSi by HNO_3_) at 400 ℃ and 850 ℃, denoted as Cu(removed)‐2DSiO_2_‐400 and Cu(removed)‐2DSiO_2_‐850, respectively. The shifts of Si 2p and O 1s peaks to higher binding energies were also observed for Cu(removed)‐2DSiO_2_‐850 in comparison to Cu(removed)‐2DSiO_2_‐400, suggesting that the higher oxidation state of Si at the higher calcining temperature is the major contributor to such shift, as the influence of Cu–O–Si was excluded in these samples by removal of Cu. However, the binding energies of Si and O for Cu(removed)‐2DSiO_2_‐*n* were still higher than Cu‐2DSiO_2_‐*n* (*n* = 400 or 850). This should originate from the contribution of Cu–O–Si species with SMSI, which keeps the 2DSi from transforming entirely to bulk SiO_2_. Nevertheless, the atomic ratios of Si to O determined by the XPS spectra approaches 1:2 for Cu‐2DSi‐850_r_, suggesting the major component of the support in Cu‐2DSi‐850_r_ is 2D SiO_2_. The chemical structures of the surface Cu species were further studied using Raman microscopy (Figure 3b). It was found that CuO was the major component in both Cu‐2DSiO_2_‐400 and Cu‐2DSiO_2_‐850. The Cu‐2DSiO_2_‐850_r_ and Cu‐2DSiO_2_‐850_ar_ samples were observed to contain Cu_2_O species, while not enough Cu_2_O signals were discovered for Cu‐2DSiO_2_‐400_r_ and Cu‐2DSiO_2_‐400_ar_. This corresponds well with the much smaller amounts of Cu^+^ in the reduced and used samples of Cu‐2DSiO_2_‐400 than Cu‐2DSiO_2_‐850 detected by Cu LMM XAES (Table S4, Supporting Information).

Since the XPS and Raman (microscope) experiments mainly characterize the surface of the catalysts, the X‐ray absorption spectroscopy (XAS) studies were further performed to study the chemical states of the integrated Cu components of the samples. The Cu K‐edge X‐ray absorption near edge structure (XANES) spectra show that the near‐edge absorption energy of Cu‐2DSiO_2_‐400 and Cu‐2DSiO_2_‐850 resemble that of CuO, while the rest samples (Cu‐2DSiO_2_‐400_r_, Cu‐2DSiO_2_‐850_r_, Cu‐2DSiO_2_‐400_ar_, and Cu‐2DSiO_2_‐850_ar_) show characteristic curves of metallic Cu (Figure 3c). Therefore, the previously seen Cu^+^ species with small amounts should only exist near the surface of Cu nanoparticles or the Cu–O–Si interface. As shown in Figure 3d, Figure S12 and Table S5 (Supporting Information), the phase‐corrected Fourier transforms of k^3^‐weighted Cu K extended X‐ray absorption fine structure (EXAFS) spectra and the fitting results of them show that the Cu–O for Cu‐2DSiO_2_‐400 locates at ≈1.97 Å, longer than that of Cu‐2DSiO_2_‐850 (≈1.91 Å). This indicates that the interaction between Cu and O for Cu‐2DSiO_2_‐850 is stronger than that for Cu‐2DSiO_2_‐400, which should account for the unique and stable Cu–O–Si species for Cu‐2DSiO_2_‐850. The lower coordination number (CN) of metallic Cu in Cu‐2DSiO_2_‐400_r_ (6.5) than that of Cu‐2DSiO_2_‐850_r_ (8) is in accordance with the smaller size of Cu nanoparticles of Cu‐2DSiO_2_‐400_r_ in comparison to Cu‐2DSiO_2_‐850_r_. The values of CN and distance of Cu‐O for Cu‐2DSiO_2_‐400_r_ and Cu‐2DSiO_2_‐850_r_ kept similar after long‐term high‐temperature reaction, suggesting the high stability of these two samples which should be ascribed from SMSI.

The CO adsorption properties of different Cu catalysts were evaluated to further reveal their chemical structures. As shown in Figure 3e, a slightly asymmetric adsorption curve was discovered for Cu‐2DSiO_2_‐400_r_ which was fitted to two distinct peaks located at 2113 and 2125 cm^–1^, corresponding to Cu^0^–CO and Cu^+^–CO, respectively.^[^ ^50^ ^]^ In contrast, the asymmetry of the CO adsorption curve for Cu‐2DSiO_2_‐850_r_ is much more apparent with the fitted Cu^0^–CO and Cu^+^–CO peaks located at 2124 and 2136 cm^–1^, respectively. The blue shift of the adsorption peaks for Cu‐2DSiO_2_‐850_r_ should be ascribed to its unique Cu–O–Si species with SMSI. The CO adsorption results indicate that Cu^+^ exists in Cu‐2DSiO_2_‐400_r_ and Cu‐2DSiO_2_‐850_r_ which could not be observed from the XRD patterns as Cu_2_O crystallites (Figure S4, Supporting Information). Therefore, the Cu^+^ species should exist on the surface of the Cu nanoparticles or the interface between Cu and 2DSiO_2_ that only occupy a small ratio of the Cu catalysts for these two samples.

H_2_ temperature‐programmed reduction (TPR) experiments were performed to determine the reducibility of these Cu catalysts (Figure 3f). The H_2_‐TPR curve with a sharp peak at 237 ℃ for Cu‐2DSiO_2_‐400 was located at a lower temperature range than the rest samples, which should be attributed to its high dispersion of Cu (small size) in comparison to the other Cu catalysts.^[^ ^51^ ^]^ Different from Cu‐2DSiO_2_‐400, asymmetric curves that can be fitted to two peaks were found for the other Cu catalysts in the TPR profiles. The split peaks should be ascribed to the reduction of smaller and larger Cu nanoparticles, respectively, in which the peak at the higher temperature range corresponds to the larger particles. The hard reducibility of Cu‐2DSiO_2_‐850 is revealed by its highest reduction temperature among the as‐prepared Cu catalysts. This indicates that the interaction between Cu and the support for Cu‐2DSiO_2_‐850 is the strongest. Therefore, the resulting unique Cu–O–Si species has led to its excellent catalytic performance.

**Figure 4:**
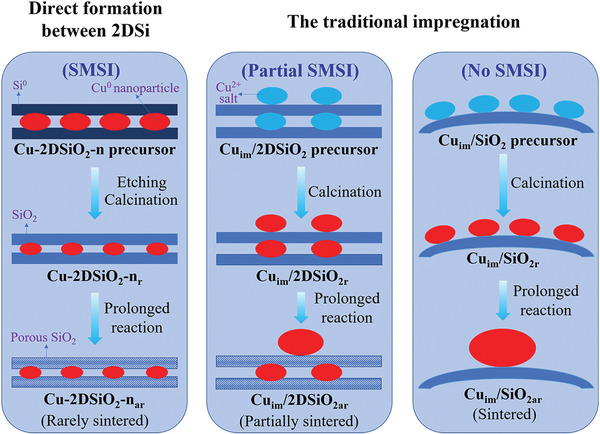
Schematic illustration of the possible evolution routes of different Cu catalysts during preparation and prolonged reaction.

Based on the above results, the possible evolution routes of the different Cu catalysts are illustrated in **Figure** **4**. The precursors of the Cu‐2DSiO_2_ based catalysts possessed an encapsulation and confined structure mainly composed of Si^0^ and Cu^0^ that can only be obtained by the exfoliation method. The encapsulation of Cu by 2DSiO_2_ could improve the stability of the Cu‐2DSiO_2_ based samples by restricting the sintering of Cu nanoparticles during high‐temperature calcination and tests. The Si^0^ and Cu^0^ species in close contact at the Cu–Si interface can easily form unique Cu–O–Si species during oxidation, generating SMSI for the Cu‐2DSiO_2_ based catalysts, in accordance with the ultrahigh stability during the prolonged test. The gradually enhanced production in the preliminary stage could also be attributed to the increased porosity of the support resulting from the corrosion of silicon oxides by the high‐temperature steam in the product gases (Figures S5, S7 and S9, Supporting Information). Notably, increasing the oxidation temperature could result in even stronger metal–support interaction which was associated with the never‐dropping conversion, and more Cu^+^ species that could lead to the improved initial conversion which corresponds well with the previous finding that Cu^+^ was the active component in Cu catalysts forming SMSI with SiO_2_ for activation and conversion of CO_2_.^[^ ^17^ ^]^ In contrast, Cu_im_/2DSiO_2r_ prepared by impregnation possessed a partial encapsulation structure with a large number of Cu nanoparticles located at the outer surface of 2DSiO_2r_. Therefore, only part of the Si^0^ and Cu^2+^ salts could form robust Cu–O–Si species with SMSI during the oxidation process. Along with the increased porosity of silicon oxides during the reaction, a rising stage in the long‐time test was also observed for Cu_im_/2DSiO_2r_. However, the sintering of Cu nanoparticles on the outer surface of 2DSiO_2_ gradually occurred during the stability experiment, leading to the reduced production rate after the initial rising stage. Finally, different from the 2DSiO_2_ based Cu catalysts, the Cu nanoparticles in Cu_im_/SiO_2_ could only locate on the outside of the silica support. The exposure of all the Cu sites led to the highest initial production rate of this sample in the long‐time testing. However, the absence of an encapsulation structure rendered the Cu nanoparticles easy to sinter during the high‐temperature reaction. Moreover, no Si^0^ exists in the Cu_im_/SiO_2_ precursor, in which the construction of SMSI is difficult. As a result, a sharp decrease in CO production was observed for Cu_im_/SiO_2r_ during the stability experiment. These results again manifest the unique advantage of the exfoliation method to prepare Cu/SiO_2_ catalysts with high performance and stability.

## 3 Conclusion

This work introduced a new and highly stable Cu catalyst supported by 2DSiO_2_ with strong metal‐support interaction. The chemical preparation procedure of this Cu‐2DSiO_2_ catalyst is environmentally benign and feasible, which has the potential to be scaled up. These Cu‐2DSiO_2_ catalysts exhibited much higher CO_2_ conversion and stability over prolonged tests than the Cu catalysts supported by 2DSiO_2_ or commercial silica prepared through the traditional impregnation method. Notably, the interaction between the active metal and the support of the Cu‐2DSiO_2_ catalysts could be enhanced by simply increasing the calcination temperature in air, which can exhibit mole (g_Cu_^–1^ h^–1^) level activity for RWGS reaction. The strategy to construct Cu‐2DSiO_2_ catalysts could provide useful guidance for the design of other‐metal‐based M‐2DSiO_2_ (M represents a specific metal) catalysts with ultrahigh activity and stability.

## 4 Experimental Section

### Materials and Chemicals

All the chemicals were used as received without further purification. Calcium silicide (CaSi_2_), ammonium hydroxide solution (NH_3_•H_2_O, 25%‐28 wt%), copper nitrate trihydrate (99.99%), and the silicon oxide support of high surface area (0.125 in pellets) were purchased from Sigma‐Aldrich, Aladdin, Macklin, and Alfa Aesar, respectively. Anhydrous copper chloride (CuCl_2_, 98.5%), ethanol (GR, ≥99.8%), and hydrochloric acid (36%–38%, analytical reagent) were purchased from Sinopharm Chemical Reagent Co., Ltd. Milli‐Q water (Millipore, 18.2 MΩ cm at 25 °C) was used in all experiments.

### Synthesis of 2DSi Supported Cu Catalysts with Various Concentrations of CuCl_2_ Precursor

CuCl_2_ was dissolved in ethanol to form a solution of *n* (*n* = 0.2, 0.2075, 0.208, 0.2085, 0.209 and 0.21) m. 80 mL of the CuCl_2_ ethanol solution were added into a round‐bottom flask. The solution was stirred at 600 rpm and heated to 60 ℃, followed by the addition of 0.8 g of CaSi_2_. The mixture was kept stirring at 60 ℃ for 3 h. After cooling to room temperature, the solid in the mixture was collected and washed with ethanol twice under centrifugation. The supernatant was removed and the precipitate was dried under vacuum.

### Synthesis of Cu‐2DSi, Cu‐2DSiO_2_‐*n*, and Cu‐2DSiO_2_‐*n*_r_

CuCl_2_ was dissolved in ethanol to form a solution of 0.2085 m. 80 mL of the CuCl_2_ ethanol solution was added into a round‐bottom flask. The solution was stirred at 600 rpm and heated to 60 ℃, followed by the addition of 0.8 g of CaSi_2_. The mixture was stirred at 60 ℃ for 3 h. After cooling to room temperature, the solid in the mixture was collected and washed with ethanol twice under centrifugation. The supernatant was removed and the precipitate was dried under vacuum. 300 mg of the dried powder (composed of small Cu nanoparticles and CuCl encapsulated by 2DSi) was mixed with 30 mL of NH_3_•H_2_O solution. The suspension was transferred to a shaking bed (400 rpm, 30 ℃) for stirring for 30 min. The mixture was then centrifuged followed by the removal of the supernatant. The precipitate (Cu‐2DSi) was washed with ethanol twice under centrifugation and then dried under vacuum. The Cu‐2DSi was then calcined at n ℃ (denoted as Cu‐2DSiO_2_‐*n*) under air, followed by reduction at 500 ℃ for 2 h under H_2_ atmosphere (denoted as Cu‐2DSiO_2_‐*n*_r_). The Cu loading of Cu‐2DSiO_2_‐*n*_r_ was 15% based on the inductively coupled plasma optical emission spectrometer (ICP‐OES) results.

### Synthesis of Cu_im_/2DSiO_2_ and Cu_im_/2DSiO_2r_

2D silicon (2DSi) was prepared through a reported recipe. 4 g of CaSi_2_ powder was dispersed in 200 mL of hydrochloric acid precooled to ‐25 ℃ under N_2_ atmosphere. The mixture was then kept at ‐20 ℃ under stirring in an inert atmosphere for 7 d. The suspension was filtered under vacuum. The filter liquor was removed and the solid product was washed with acetone by filtration under vacuum. The feed ratio of the Cu precursor and the 2DSi support was calculated based on the loading amount of Cu for Cu‐2DSiO_2_‐*n*_r_. To be specific, 0.1673 g of Cu(NO_3_)_2_•3H_2_O was dissolved in 20 mL of ethanol under stirring. 0.25 g of the 2DSi powder was added to the solution and kept under stirring at 80 ℃ until the disappearance of liquid. The powders were dried under vacuum and then calcined at 850 ℃ for 2 h in air, and the corresponding sample was denoted as Cu_im_/2DSiO_2_. The Cu_im_/2DSiO_2_ sample was then reduced at 500 ℃ for 2 h under H_2_ atmosphere, and the obtained catalyst was denoted as Cu_im_‐2DSiO_2r_.

### Synthesis of Cu_im_/SiO_2_ and Cu_im_/SiO_2r_

The feed ratio of the Cu precursor and the SiO_2_ support was calculated based on the loading amount of Cu for Cu‐2DSiO_2_‐n_r_. To be specific, 0.3377 g of Cu(NO_3_)_2_•3H_2_O was dissolved in 20 mL of ethanol under stirring. 0.5044 g of the silicon oxide support (grinded to penetrate through an 80‐mesh sieve) was added to the solution and kept under stirring at 80 ℃ until the disappearance of liquid. The powders were dried under vacuum and then calcined at 850 ℃ for 2 h in air, and the corresponding sample was denoted as Cu_im_/SiO_2_. The Cu_im_/SiO_2_ sample was then reduced at 500 ℃ for 2 h under H_2_ atmosphere, and the obtained catalyst was denoted as Cu_im_/SiO_2r_.

### Thermocatalytic Tests

Flow reactor studies for CO_2_ hydrogenation under atmospheric pressure were carried out in a quartz tube reactor (CEL‐GPPCM, Beijing China Education AU‐LIGHT Co., Ltd., Figure S13, Supporting Information). The oxidized sample (Cu‐2DSiO_2_‐400, Cu‐2DSiO_2_‐850, Cu_im_/2DSiO_2_ or Cu_im_/SiO_2_, grinded to penetrate through an 80‐mesh sieve) was reduced in the flow reactor in situ at 500 ℃ under H_2_ for 2 h. The temperature was kept at 500 ℃ and the flowing gas in the reactor changed from H_2_ to the reaction atmosphere (CO_2_: H_2_: N_2_ = 5: 5: 5 mL min^‐1^) after the reduction process. The testing longed for 54 h for all the Cu catalysts. For Cu‐2DSiO_2_‐400 and Cu‐2DSiO_2_‐850, the CO production rates at various temperatures were also collected by reducing the reaction temperature after the long‐time testing to acquire the Arrhenius plot (Table S2, Supporting Information). The concentration of gas products was analyzed online by a gas chromatograph (Agilent 8860) equipped with a flammable ionization detector (FID) and a thermal conductive detector (TCD). Since CO was the only product for all the Cu catalysts, the conversion of CO_2_ ($X_{CO_{2}}$) was defined as:

(1) $$X_{CO_{2}\;}=\;\;\frac{F_{\mathrm{CO}(\mathrm{outlet})}}{F_{CO_{2}\left(\mathrm{inlet}\right)}}=\frac{\frac{C_{\mathrm{CO}(\mathrm{outlet})}}{C_{N_{2}\left(\mathrm{outlet}\right)}}}{\frac{C_{CO_{2}\left(\mathrm{inlet}\right)}}{C_{N_{2}\left(\mathrm{inlet}\right)}}}\;$$

where *F* is the flow rate of reactants or products (mol min^‐1^), *C* is the concentration of reactants or products (%).

### The H_2_ TPR Experiments

20 mg of the Cu catalyst was put inside a flow reactor and heated to 400 ℃ at a heating rate of 10 ℃ min^‐1^ under Ar (20 mL min^‐1^). The catalyst was kept at 400 ℃ for 30 min under Ar and then cooled to room temperature. The flowing gas changed from Ar to 10%H_2_/Ar. After that, the reactor was heated to 800 ℃ at a heating rate of 10 ℃ min^‐1^. The signal of the gas product was recorded online with a TCD (Agilent).

### The CO Adsorption Experiments

In situ diffuse reflection infrared Fourier transform spectroscopy (DRIFTS) experiments of CO adsorption were performed with an in situ DRIFTS reaction cell (Hefei In situ Technology Co., LTD.) and an FTIR spectrometer (Thermo Nicolet iS50). Approximately 20 mg of the as‐reduced catalysts (grinded to penetrate through an 80‐mesh sieve) were packed into the in situ cell. The catalyst was further reduced in situ in the cell at 400 ℃ under H_2_ for 0.5 h. The flowing gas was changed from H_2_ to Ar and the reactor was cooled to room temperature. The inlet flow was switched to CO of 20 mL min^‐1^. The adsorption species on the surface of catalysts were detected online.

### Characterization

TEM images, STEM images, and EDX mappings were obtained with an FEI Talos F200X (200 kV) transmission electron microscope. X‐ray absorption spectroscopy experiments were performed at the 4B9A beamline of the Beijing Synchrotron Radiation Facility (BSRF). X‐ray photoelectron spectroscopy (XPS) and X‐ray induced Auger electron spectroscopy (XAES) were obtained using a Thermo Scientific K‐Alpha Scanning ESCA Microprobe instrument (physical Electronics) equipped with an Al K*α* X‐ray source (*hν* = 1486.6 eV) and binding energies referenced to C1s (284.8 eV). The metal content of different samples was measured by an Inductively coupled plasma optical emission spectrometer (ICP‐OES) (i CAP Pro X, Thermofisher). X‐ray diffraction (XRD) was measured on an XRD‐700 X‐ray diffractometer from Shimadzu. Raman spectra were obtained with a Raman spectrometer (Horiba LabRAM HR Evolution) equipped with a 532‐nm‐laser excitation source.

### Statistical Analysis

The particle size of Cu for different Cu samples was measured by nanomeasurement: at least 100 particles were analyzed for each sample. Two to four data points were recorded for each testing condition in thermocatlytic CO_2_ hydrogenation and the CO rate under each condition is the average value of the corresponding points.

## Conflict of Interest

The authors declare no conflict of interest.

## Author Contributions

S.W. and K.F. contributed equally to this work. W.S., S.W., K.F., B.Y., and G.A.O conceived and designed the experiments. S.W. carried out and analyzed the synthesis, catalysis, XRD, XPS, and ICP experiments. K.F. carried out the XAS, H_2_ TPR, Raman experiments and analyzed the XAS results. D.Z. carried out the TEM and CO adsorption experiments. M.X. and C.Z. carried out the TEM characterization. K.F., B.Y., D.Y., and L.H. provided suggestions for the manuscript. W.S., D.Y., B.Y., L.H. provided the financial or equipment support for this work. S.W., W.S., and G.A.O. wrote the paper. All authors commented on the final manuscript.

## Supporting information

Supporting Information

## Data Availability

The data that support the findings of this study are available from the corresponding author upon reasonable request.
