# Supplementary material for: Stable Cu Catalysts Supported by Two‐dimensional SiO2 with Strong Metal–Support Interaction
Source: Adv Sci (Weinh). 2022 Jan 25;9(9):2104972. doi: 10.1002/advs.202104972 (PMC8948561; doi:10.1002/advs.202104972)
Supplement: Supplementary file 1 — Supporting Information [file ADVS-9-2104972-s001.pdf]

ADVANCED SCIENCE
Open Access

Supporting Information

for *Adv. Sci.*, DOI: 10.1002/advs.202104972

Stable Cu Catalysts Supported by Two-dimensional SiO_2 with Strong Metal-support Interaction

Shenghua Wang, Kai Feng, Dake Zhang, Deren Yang, Mengqi Xiao, Chengcheng Zhang, Le He, Binhang Yan, Geoffrey A. Ozin, and Wei Sun**

Supporting Information

Stable Cu Catalysts Supported by Two-dimensional SiO_2 with Strong Metal-support Interaction

Shenghua Wang[†], Kai Feng[†], Dake Zhang, Deren Yang, Mengqi Xiao, Chengcheng Zhang, Le He, Binhang Yan, Geoffrey A. Ozin, and Wei Sun**

[†]S. Wang and K. Feng contributed equally to this work.

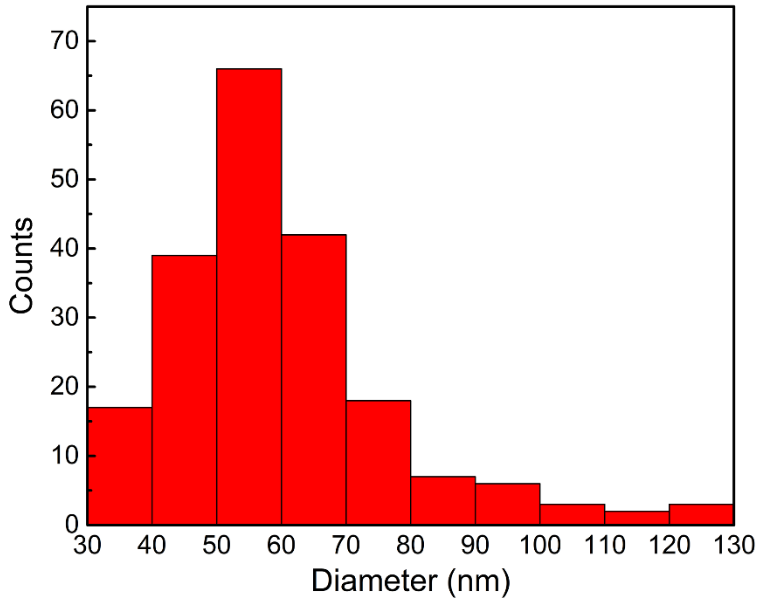

Figure S1. Copper size distribution of 2DSi supported large Cu nanoparticles in Fig. 1c.

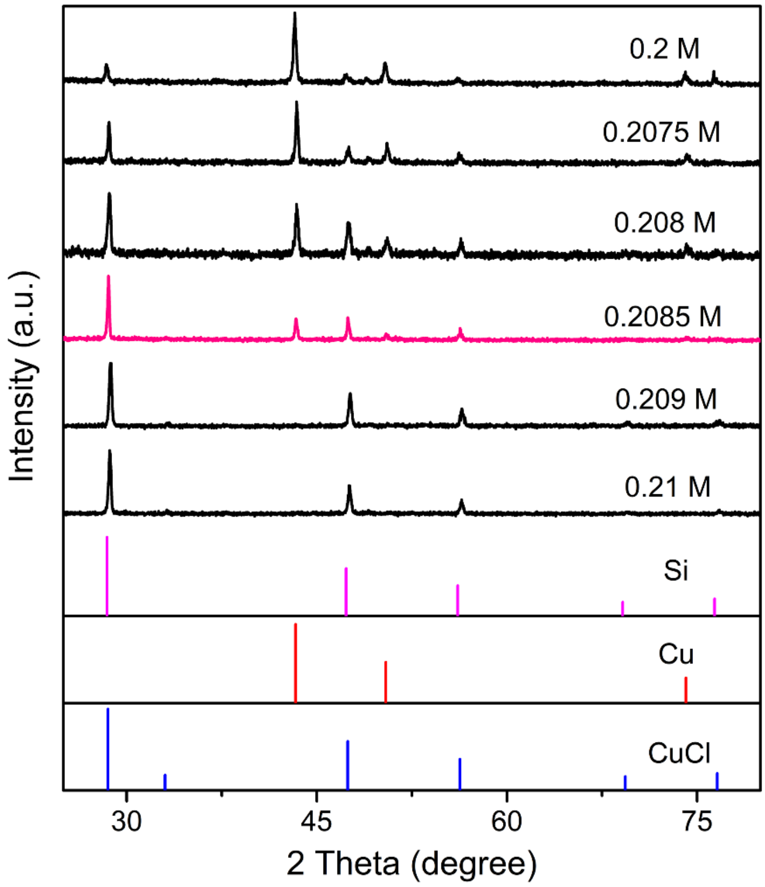

Figure S2. XRD patterns of 2DSi-encapsulated-copper samples with different $CuCl_2$ concentrations, the standard Si (JCPDS 27-1402), the standard Cu (JCPDS 04-0836) and the standard CuCl (JCPDS 06-0344).

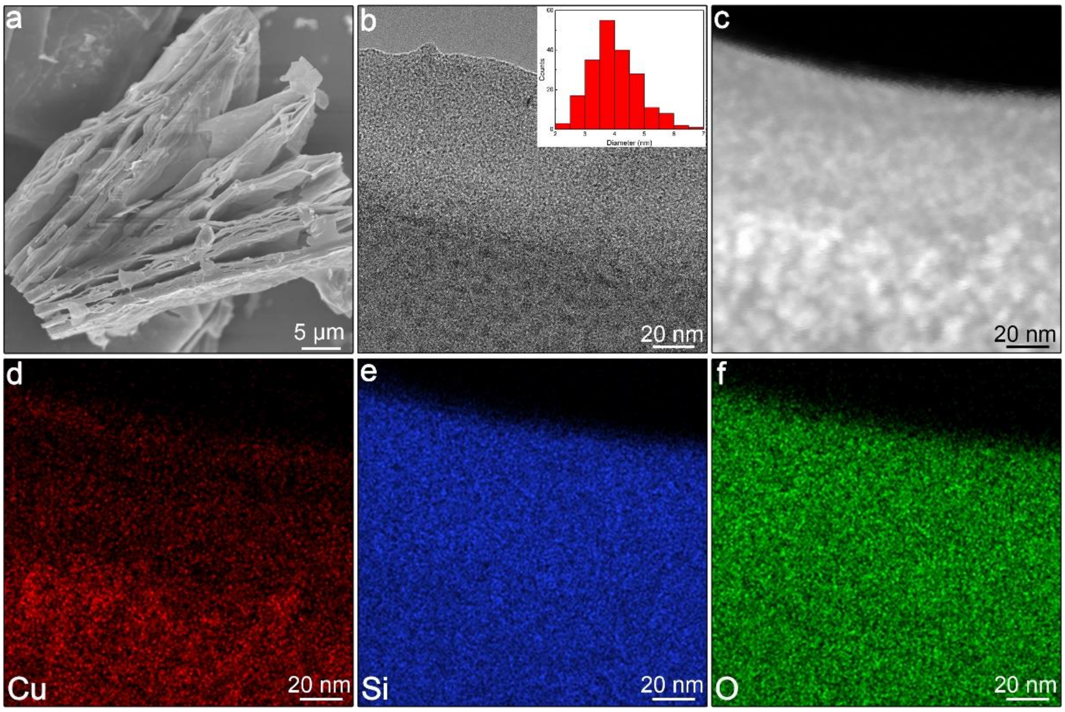

Figure S3. **(a)** SEM image, **(b)** TEM image and **(c-f)** EDS mappings of Cu-2DSiO_2-400. The inset in (b) is the copper size distribution of Cu-2DSiO_2-400.

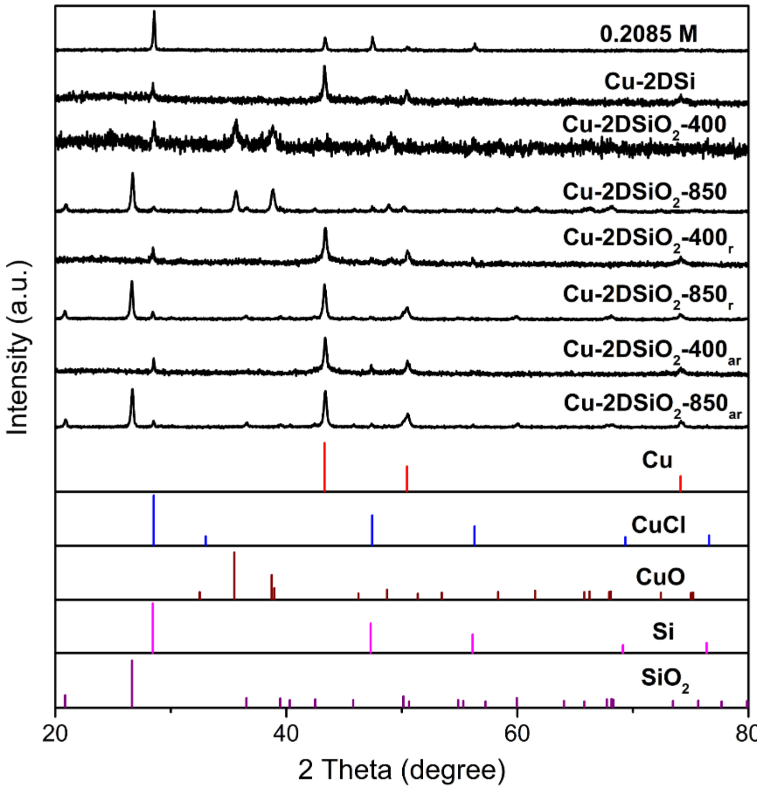

Figure S4. XRD patterns of Cu samples prepared with the $CuCl_2$ concentration of 0.2085 M treated under different conditions, the standard Cu (JCPDS 04-0836), the standard CuCl (JCPDS 06-0344), the standard CuO (JCPDS 45-0937), the standard Si (JCPDS 27-1402), and the standard SiO_2 (JCPDS 46-1045).

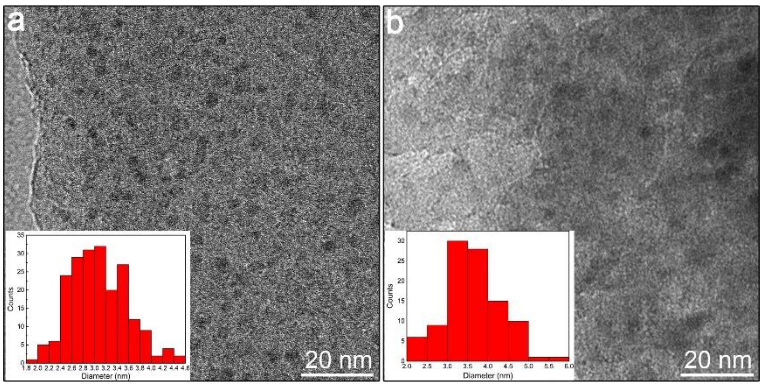

Figure S5. TEM images of **(a)** Cu-2DSiO$_2$-400$_r$ and **(b)** Cu-2DSiO$_2$-400$_{ar}$. The insets in (a) and (b) are the copper size distributions of Cu-2DSiO$_2$-400$_r$ and Cu-2DSiO$_2$-400$_{ar}$, respectively.

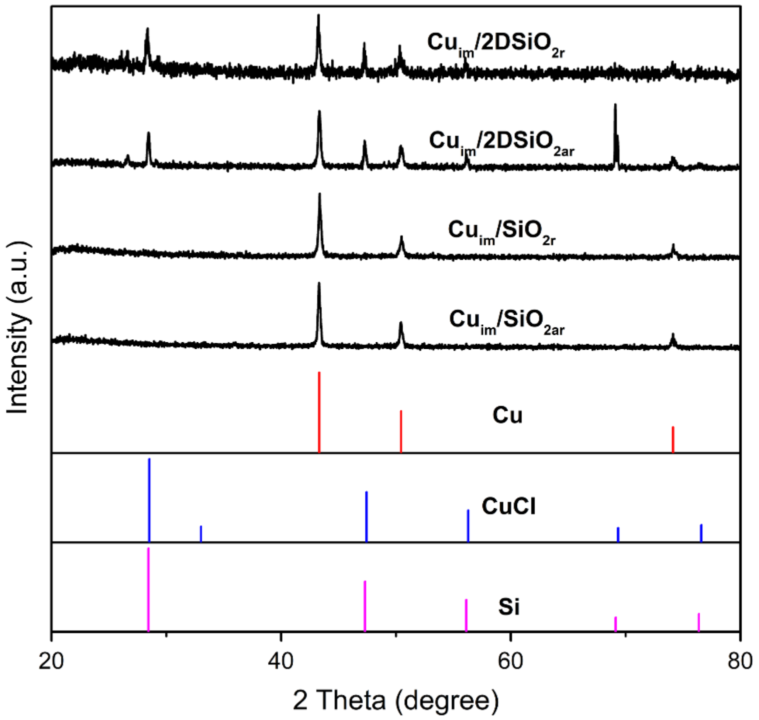

Figure S6. XRD patterns of the Cu catalysts prepared through impregnation, the standard Cu (JCPDS 04-0836), the standard CuCl (JCPDS 06-0344), and the standard Si (JCPDS 27-1402).

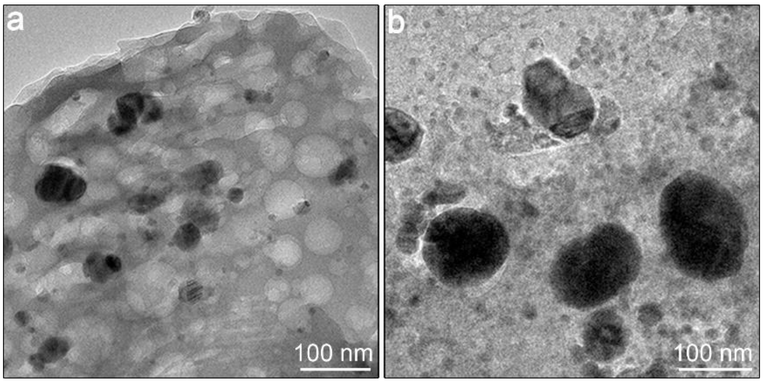

Figure S7. TEM images of **(a)** $Cu_{im}/2DSiO_{2r}$ and **(b)** $Cu_{im}/2DSiO_{2ar}$.

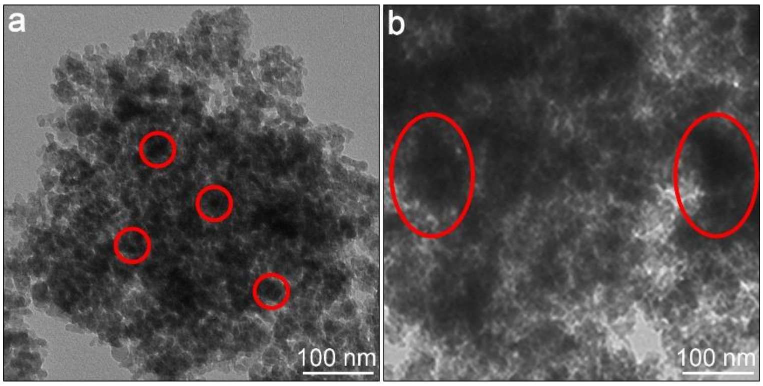

Figure S8. TEM images of **(a)** Cu_{im}/SiO_{2r} and **(b)** Cu_{im}/SiO_{2ar}.

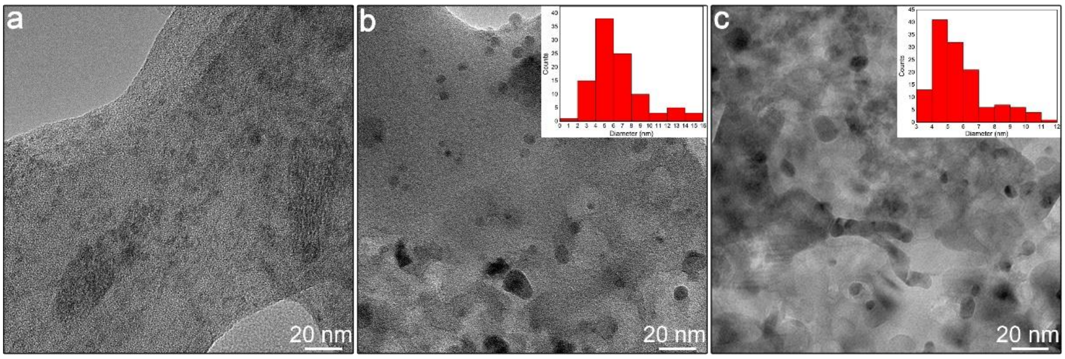

Figure S9. TEM images of **(a)** Cu-2DSiO$_2$-850, **(b)** Cu-2DSiO$_2$-850$_r$ and **(c)** Cu-2DSiO$_2$-850$_{ar}$. The insets in (b) and (c) are the copper size distributions of Cu-2DSiO$_2$-850$_r$ and Cu-2DSiO$_2$-850$_{ar}$, respectively.

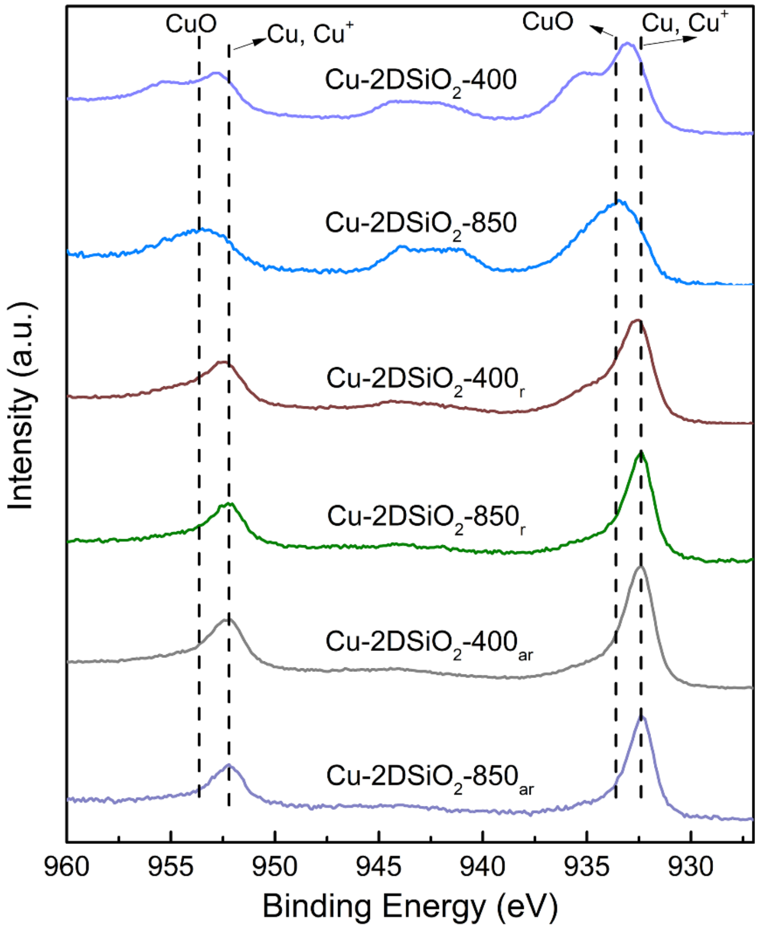

Figure S10. Cu 2p XPS spectra of different Cu catalysts.

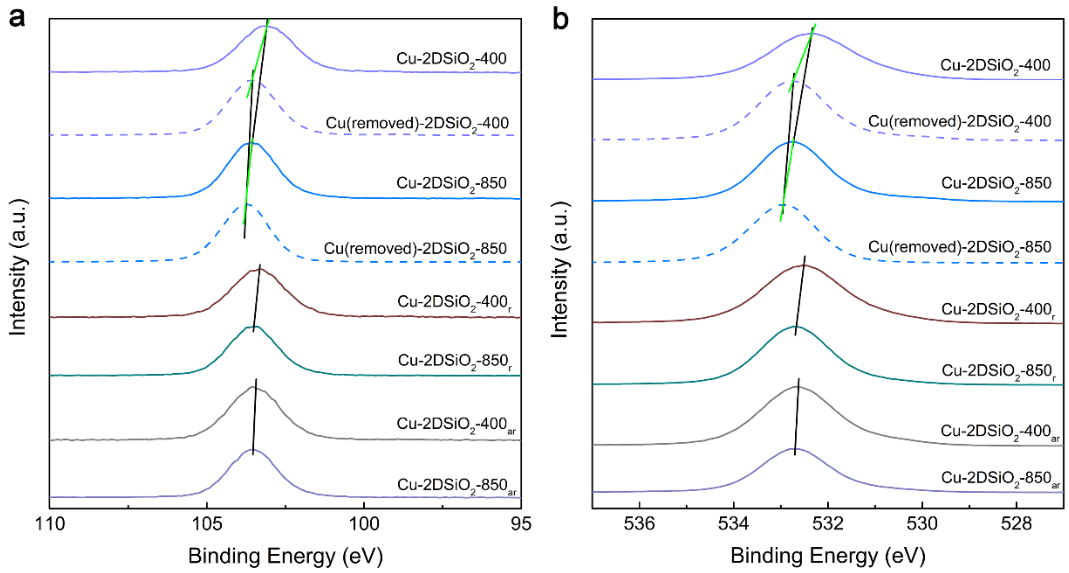

Figure S11. (a) Si 2p and (b) O 1s XPS spectra of different Cu catalysts.

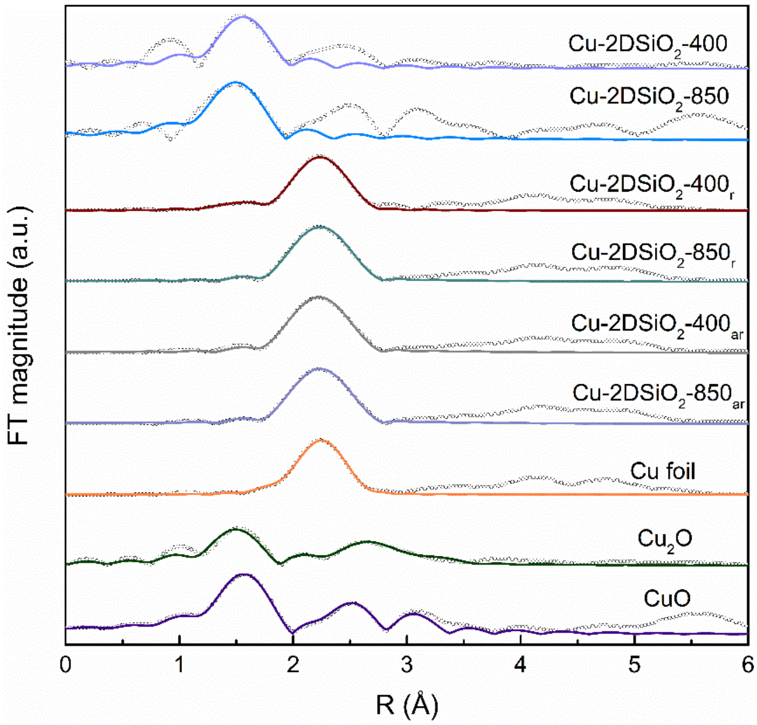

Figure S12. (a) Cu K-edge EXAFS fitting results of different Cu catalysts. The black open circles show the original data of the EXAFS spectra, while the lines of other colours are the fitting results of the spectra.

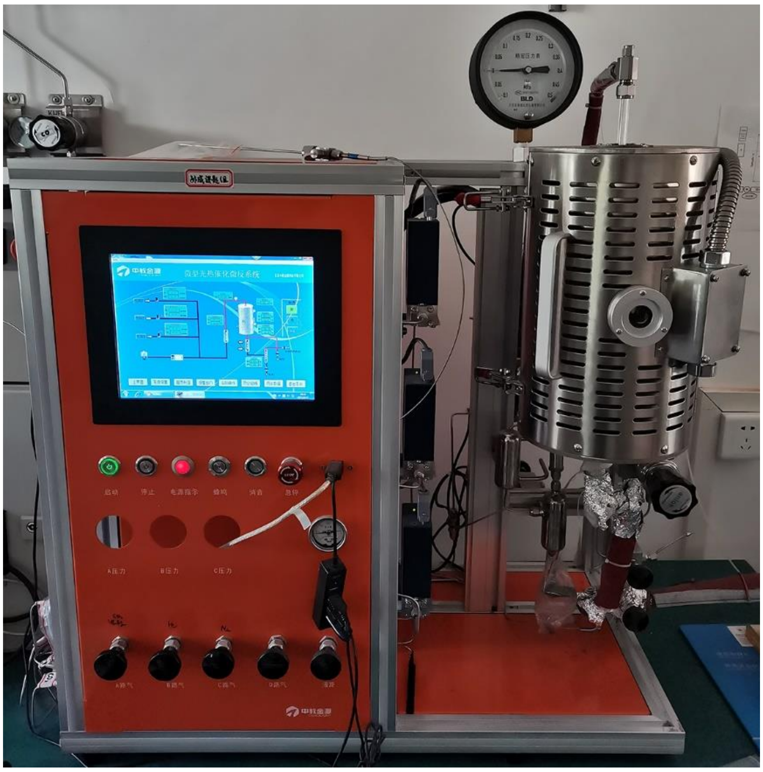

Figure S13. The flow reactor used in this work.

Table S1. Comparison of the catalytic performance of our catalyst and the other reported Cu/SiO_2 systems.

Reference	Catalyst	Space velocity (mL g_{cat}^{-1} h^{-1})	CO_2: H_2	T (˚C)	CO rate (mmol g_{Cu}^{-1} h^{-1})	CO_2 conv (%)
This work	Cu-2DSiO_2-850_r	18840	1:4	500		38%
This work[a]	Cu-2DSiO_2-850_r	3000000	1:2	550	4442	<10%
[1]	CuNi/SiO_2	4200	1:4	500		~43%
[1]	Cu/SiO_2	4200	1:4	500		~29%
[2]	CuSiO-I	3000000	1:2	550	3798	<10%
[3]	CuSiO/CuO_X	60000	1:4	500		25%
[4]	K doped Cu/SiO_2	300000	1:1	500		3.4%
[5]	ALE-Cu/SiO_2	120000	1:1	500		~4.8%

[a]1 mg of the Cu-2DSi-850 catalyst was diluted by 40 mg of inert SiO_2. The rate was determined at the 10th hour after the temperature reached 550 ˚C.

Table S2. CO rates at various temperatures for Cu-2DSiO_2-400_r and Cu-2DSiO_2-850_r.

**Cu-2DSiO_2-400_r**		**Cu-2DSiO_2-850_r**	
**Temperature**	**CO rate***	**Temperature**	**CO rate***
442 ˚C	12.73	433 ˚C	12.19
452 ˚C	15.11	443 ˚C	14.68
462 ˚C	17.84	453 ˚C	17.54
472 ˚C	20.27	463 ˚C	20.56
482 ˚C	23.83	473 ˚C	23.97

*The unit of the CO rate is $mmol \cdot g^{-1} \cdot h^{-1}$.

Table S3. Comparison of the stability of our catalyst and the reported Cu based catalysts for RWGS.

Reference	Catalyst	Space velocity (mL g_{cat}^{-1} h^{-1})	CO_2: H_2	T (˚C)	Time (h)	Activity lost
This work	Cu-2DSiO$_2$-850$_r$	18000	1:1	500	54	-36%[a]
This work	Cu-2DSiO$_2$-400$_r$	18000	1:1	500	54	-26%[a]
This work	Cu$_{im}$/2DSiO$_{2r}$	18000	1:1	500	54	-27%[a]
This work	Cu$_{im}$/SiO$_{2r}$	18000	1:1	500	54	53%
[6]	Cu/Al_2O_3	12000	1:9	500	1	~30%
[7]	CeO_2@Cu-A	360000	1:4	400	15	~13%
[8]	0.75Fe0.25Cu	60000	1:4	450	25	~10%
[9]	CuAlSi-900	15000	1:3	450	25	~25%
[10]	Cu(1)/FAU	7500	1:1	500	20	~13%

[a]*The negative value means that the final activity is even higher than the initial activity in the long-term test.*

Table S4. Calculated Cu^+ atomic ratios (%) of different Cu catalysts from the Cu LMM XAES results.

Sample	Cu^+ atomic ratio (%)
Cu-2DSiO$_2$-400	21.3
Cu-2DSiO$_2$-400$_r$	20.4
Cu-2DSiO$_2$-400$_{ar}$	23.7
Cu-2DSiO$_2$-850	33.9
Cu-2DSiO$_2$-850$_r$	29.2
Cu-2DSiO$_2$-850$_{ar}$	34.2

Table S5. Structure parameters extracted from the EXAFS fitting of Cu K-edge.

Catalysts	Path	CN	Distance/Å	$\sigma^2/10^{-3}$ Å^2	$\triangle E_0$/eV	R^2/%
**Cu-2DSiO$_2$-400**	Cu-O (CuO)	2.6±0.4	1.97±0.03	2.2	2.02	0.6
**Cu-2DSiO$_2$-850**	Cu-O (CuO)	3.7±1.1	1.91±0.03	4.5	-0.04	1.5
**Cu-2DSiO$_2$-400$_r$**	Cu-O (CuO)	0.6±0.1	1.89±0.02	9.1	-0.02	0.7
	Cu-Cu (Cu)	6.5±0.2	2.54±0.01	1.7	-0.07	
**Cu-2DSiO$_2$-850$_r$**	Cu-Cu (Cu)	8.0±0.6	2.54±0.01	8.2	-0.02	0.5
**Cu-2DSiO$_2$-400$_{ar}$**	Cu-Cu (Cu)	6.5±0.7	2.54±0.01	9.0	-0.02	1.2
**Cu-2DSiO$_2$-850$_{ar}$**	Cu-Cu (Cu)	8.6±0.6	2.54±0.01	8.6	-0.02	0.4
**Cu foil**	Cu-Cu (Cu)	12	2.56			
**CuO**	Cu-O (CuO)	4	1.95			
**Cu$_2$O**	Cu-O (Cu$_2$O)	2	1.86			

References

[1] A. Kumar, A. A. A. Mohammed, M. A. H. S. Saad, M. J. Al-Marri, *Int. J. Energy Res.* **2021**, https://doi.org/10.1002/er.6586.

[2] R. Jin, J. Easa, C. P. O'Brien, *ACS Appl. Mater. Interfaces* **2021**, *13*, 38213.

[3] Y. Yu, R. Jin, J. Easa, W. Lu, M. Yang, X. Liu, Y. Xing, Z. Shi, *Chem. Commun.* **2019**, *55*, 4178.

[4] C.-S. Chen, W.-H. Cheng, S.-S. Lin, *Appl. Catal. A-Gen.* **2003**, *238*, 55.

[5] C. S. Chen, J. H. Lin, J. H. You, C. R. Chen, *J. Am. Chem. Soc.* **2006**, *128*, 15950.

[6] C.-S. Chen, W.-H. Cheng, S.-S. Lin, *Catal. Lett.* **2000**, *68*, 45.

[7] B. Lu, Y. Xu, Z. Zhang, F. Wu, X. Li, C. Luo, L. Zhang, *J. CO2 Util.* **2021**, *54*, 101757.

[8] L. Yang, L. Pastor-Pérez, J. J. Villora-Pico, A. Sepúlveda-Escribano, F. Tian, M. Zhu, Y.-F. Han, T. Ramirez Reina, *ACS Sustain. Chem. Eng.* **2021**, *9*, 12155.

[9] A. M. Bahmanpour, B. P. Le Monnier, Y.-P. Du, F. Héroguel, J. S. Luterbacher, O. Kröcher, *Chem. Commun.* **2021**, *57*, 1153.

[10] A. Okemoto, M. R. Harada, T. Ishizaka, N. Hiyoshi, K. Sato, *Appl. Catal. A-Gen.* **2020**, *592*, 117415.
